# EGF Functionalized Polymer-Coated Gold Nanoparticles Promote EGF Photostability and EGFR Internalization for Photothermal Therapy

**DOI:** 10.1371/journal.pone.0165419

**Published:** 2016-10-27

**Authors:** Catarina Oliveira Silva, Steffen B. Petersen, Catarina Pinto Reis, Patrícia Rijo, Jesús Molpeceres, Ana Sofia Fernandes, Odete Gonçalves, Andreia C. Gomes, Isabel Correia, Henrik Vorum, Maria Teresa Neves-Petersen

**Affiliations:** 1 Research Center for Biosciences & Health Technologies, Universidade Lusófona, Lisboa, Portugal; 2 Department of Biomedical Sciences, Faculty of Pharmacy, University of Alcalá, Alcalá de Henares, Spain; 3 Medical Photonics Lab, Department of Health Science and Technology, Faculty of Medicine, Aalborg University, Aalborg, Denmark; 4 IBEB, Biophysics and Biomedical Engineering, Faculty of Sciences, University of Lisbon, Lisbon, Portugal; 5 iMed.ULisboa, Instituto de Investigação do Medicamento, Faculdade de Farmácia, Universidade de Lisboa, Lisboa, Portugal; 6 CBMA (Centre of Molecular and Environmental Biology), University of Minho, Campus de Gualtar, Braga, Portugal; 7 CFUM (Centre of Physics of University of Minho), Department of Physics, University of Minho, Campus de Gualtar, Braga, Portugal; 8 Centro de Química Estrutural, Instituto Superior Técnico, Universidade de Lisboa, Lisbon, Portugal; 9 Department of Ophthalmology, Aalborg University Hospital, Aalborg, Denmark; 10 Department of Clinical Medicine, Aalborg University Hospital, Aalborg, Denmark; Helsingin Yliopisto, FINLAND

## Abstract

The application of functionalized nanocarriers on photothermal therapy for cancer ablation has wide interest. The success of this application depends on the therapeutic efficiency and biocompatibility of the system, but also on the stability and biorecognition of the conjugated protein. This study aims at investigating the hypothesis that EGF functionalized polymer-coated gold nanoparticles promote EGF photostability and EGFR internalization, making these conjugated particles suitable for photothermal therapy. The conjugated gold nanoparticles (100–200 nm) showed a plasmon absorption band located within the near-infrared range (650–900 nm), optimal for photothermal therapy applications. The effects of temperature, of polymer-coated gold nanoparticles and of UVB light (295nm) on the fluorescence properties of EGF have been investigated with steady-state and time-resolved fluorescence spectroscopy. The fluorescence properties of EGF, including the formation of Trp and Tyr photoproducts, is modulated by temperature and by the intensity of the excitation light. The presence of polymeric-coated gold nanoparticles reduced or even avoided the formation of Trp and Tyr photoproducts when EGF is exposed to UVB light, protecting this way the structure and function of EGF. Cytotoxicity studies of conjugated nanoparticles carried out in normal-like human keratinocytes showed small, concentration dependent decreases in cell viability (0–25%). Moreover, conjugated nanoparticles could activate and induce the internalization of overexpressed Epidermal Growth Factor Receptor in human lung carcinoma cells. In conclusion, the gold nanoparticles conjugated with Epidermal Growth Factor and coated with biopolymers developed in this work, show a potential application for near infrared photothermal therapy, which may efficiently destroy solid tumours, reducing the damage of the healthy tissue.

## Introduction

Nanocarriers with improved characteristics, such as size, shape and plasmonic surface properties are selected for photonic therapeutic applications for cancer treatment [1]. One of the most studied and potential application is the near-infrared (NIR) photothermal therapy based on gold-nanoparticle-mediated hyperthermia and, consequently, protein denaturation and tissue necrosis [1]. As multifunctional system, nanocarriers are further functionalized with small targeting peptides [2]. Therefore, the success of the photothermal therapy depends on the therapeutic efficiency and biocompatibility of the system, but also on the stability and biorecognition properties of the conjugated biomolecule.

Bio-functionalization of nanoparticles with EGF has been applied to specific targeting cancer cells, which overexpress EGFR and therefore, with ample interest for photothermal cancer treatment. EGF offers many advantages for this type of pharmaceutical application: 1) EGF is smaller (53 amino acids; MW: 6 kDa) than antibodies or other EGFR specific ligands used for the same purpose; 2) unlike EGF, antibodies can trigger severe immune response leading to cytotoxicity [1]; 3) EGF has three SS bonds, three Trp and five Tyr and hydrophobic residues, all suitable for interactions with nanocarriers [3]; and 4) EGF is stable at physiological conditions and neutral pH since its pI value is around 4.55, conferring the peptide a negative charge at pH > 7 [4]. Fourier Transform Infrared (FT-IR) studies also showed that EGF presents a thermal unfolding at pH 7.2 that starts at 40°C, with the transition midpoint at 55.5°C and complete denaturation is observed above 76°C [5]. Another study evaluated the application of EGF for skin patches, showing the resistance of this peptide to temperature (T_m_ ~ 79°C) [6]. However, the potential physiological activation and stimulation of cancer growth has hindered the use of EGF as targeting peptide in drug delivery systems, which is dependent on the release of the anticancer drug [7]. When conjugated to metallic nanoparticles, EGF promotes a rapid internalization into cancer cells [8], and cancer destruction can be achieved by injecting the light-absorbing nanoparticles locally and applying the laser-mediated hyperthermia directly into the tumour. Therefore, it is our aim to use EGF-conjugated HAOA-coated gold nanoparticles with plasmon absorption band located in the near-infrared (NIR) range (i.e., 650–900 nm), for photothermal therapy and local hyperthermia, without damage to the surrounding tissues [9]. This study describes the behaviour of EGF when exposed to temperature, UVB light (295 nm) and quenchers, such as gold nanoparticles coated by hyaluronic and oleic acids (HAOA). The use of oleic acid (OA) and polymers like hyaluronic acid (HA) can further promote the interaction and entrapment of EGF onto gold nanoparticles, independently of the pI and pH of the solution, as previously reported [10]. In addition, HA is described to be an excellent fluorescence quencher [11] and to give structural stability to small proteins [12]. OA is also described as a good protein fluorescence quencher [13]. The presence of quenchers shortens the fluorescence lifetimes and may confer protection against photochemistry. Therefore, we have investigated if the presence of gold nanoparticles coated with OA and HA protected the attached EGF from UV wavelengths normally used to trigger protein fluorescence, such as 295 nm.

UV excitation of proteins causes protein conformational changes upon excitation of the aromatic residues, i.e., tryptophan (Trp), tyrosine (Tyr) and phenylalanine (Phe). Three main photoproducts are kynurenine (Kyn, a photoproduct of Trp), N—formylkynurenine (NFK, a photoproduct of Trp) and dityrosine (DT, a photoproduct of Tyr) [14–18]. Furthermore, UV excitation of the side chains of aromatic residues induces the disruption of disulphide (SS) bonds, mediated by an electron transfer process, leading to the formation of a transient disulphide electron adduct and to changes in the fluorescence quantum yield of proteins [16,17]. The effect of UV light on the structure and function of key medically relevant proteins, such as Epidermal Growth Factor Receptor (EGFR) [19], insulin [14] and plasminogen [15] has been reported.

The present study reports the time dependent effect of continuous 295 nm excitation of free EGF on the peptide’s fluorescence emission intensity, as a function of irradiance level (power/unit area) and temperature. Trp was selected as an intrinsic molecular probe and SYPRO^®^ Orange was used as an extrinsic molecular probe in order to monitor protein conformational changes [20]. The formation of photoproducts, NKF, Kyn and DT, has been monitored. Moreover, the expected protective effect provided by HAOA-coated gold nanoparticles against 295 nm-induced photochemistry on EGF was investigated by fluorescence spectroscopy and, structurally, by circular dichroism spectroscopy. Binding of EGF and EGF-conjugated HAOA-coated gold nanoparticles to EGFR, present on the cell membrane of A549 human lung carcinoma cells, was monitored using confocal fluorescence microscopy and cytotoxicity assays (MTT) were carried out in non-cancerous human immortalized keratinocytes, HaCaT cell line.

## Materials and Methods

### Materials

Gold (III) chloride trihydrate (HAuCl_4_) (PubChem ID: 24895143; Product number: G4022), L-ascorbic acid (L-AA) (PubChem ID: 24891246; Product number: A7506), silver nitrate (AgNO_3_) (PubChem ID: 24852543; Product number: S0139), hyaluronic acid (HA) sodium salt from *Streptococcus equi* (MW: 7,000–250,000 g.mol^-1^) (PubChem ID: 24878223; Product number: 53747), oleic acid (OA) (MW: 282.46 g.mol^-1^) (PubChem ID: 24886786; Product number: 75090) were all supplied by Sigma-Aldrich (Steinheim, Germany). Recombinant Human Epidermal Growth Factor (EGF) (PubChem ID: 62253638), Alexa Fluor^®^ 647 and SYPRO^®^ Orange Protein Gel Stain (5,000X Concentrate in DMSO) was purchased from Life Technologies as molecular probes for confocal microscopy and protein conformational studies. Primary mouse monoclonal antibody anti-EGFR neutralizer antibody LA1 was obtained from Millipore (05–101). The water used for buffer preparation was purified through a Millipore system. Thiazolyl Blue Tetrazolium Bromide (MTT), Fetal Bovine Serum (FBS), puromycin and penicillin/streptomycin were supplied by Sigma-Aldrich (Steinheim, Germany), as of cell culture grade. Dulbecco's Modified Eagle's medium (DMEM) was supplied by Biowest (Nuaillé, France) and DMSO was supplied by Merck (Darmstadt, Germany).

### Preparation of EGF stock solution and EGF-conjugated gold nanoparticles

A 2.5 μM (16.5 μg/mL) stock solution of EGF was prepared in 2 mM Phosphate Buffer Saline (PBS) at pH 7.4. In order to prepare EGF-conjugated gold nanoparticles, the EGF stock solution at 2.5 μM was mixed with the gold nanoparticles solution (0.22 mM) and hyaluronic acid-oleic acid (HAOA) solution (1 mg/mL), at a 1:1 (v/v) ratio. The reaction mixture was kept for 30 min at room temperature and, then, left overnight at 4°C protected from the light. Gold nanoparticles were produced based on the addition of an aqueous extract of *Plectranthus saccatus* (10 mg/ mL) as the main reducing and capping agent [21]. The aqueous plant extract was used in alternative to cetyl trimethylammonium bromide (CTAB), and prepared according to the procedure described by Rijo et al. (2014), using a microwave method [22]. The nanoparticles suspension was centrifuged twice at 500 x g for 20 min in a FV2400 Microspin (BioSan, Riga, Latvia) to remove unbound peptides. The pellet was re-suspended in PBS buffer (pH 7.4). EGF stock solution was stored at -20°C until further use.

### EGF structure analysis and gold nanoparticles structure design

**Fig 1:**
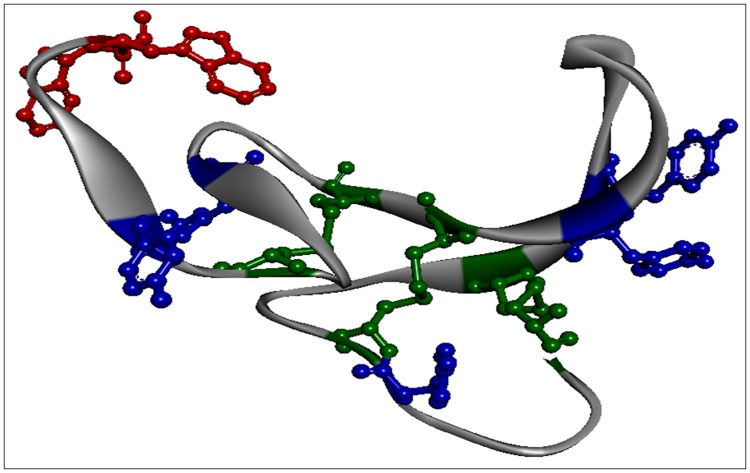
Molecular structure of EGF (chain B) according to (1JL9.pdb). Aromatic residues are represented by different colors: Trp (red), Tyr (blue), Cys (green).

Crystallographic data used for the display of the 3D protein structure (Fig 1) was extracted from 1JL9.pdb (3D structure of EGF, chain B), using Discovery Studio 4.1 (Accelrys Software, San Diego, CA, USA). Distances between protein residues were obtained by using the monitor tool in the program (Table 1). Adobe Illustrator CS5 (Adobe Systems Software Ireland Ltd.) was used in order to graphically display the EGF-conjugated HAOA-coated gold nanoparticles.

**Table 1:** Shortest spatial distances between disulphide (SS) bonds and aromatic residues (tryptophan and tyrosine) in EGF chain B (1JL9.pdb). The shortest distances (< 12 Å) between atoms of each pair of elements (Trp, Tyr and disulphide bonds) were considered. For Trp and Tyr residues, only atoms belonging to the indole and benzene rings were considered, and for SS bonds one of the SG atoms. (W = Trp; Y = Tyr; PDB atomtype descriptor used is given in parentheses).

Disulphide Bond	Aromatic Residue	Distance (Å)
**C6-C20**	Y13 (CD1)	5.5
	Y22 (CG)	9.2
	Y29 (CD1)	4.8
**C14-C31**	Y13 (CD1)	4.4
	Y29 (CD1)	9.5
	Y37 (CD2)	9.7
	Y44 (CD2)	11.8
**C33-C42**	Y13 (CD1)	7.9
	Y37 (CG)	5.7
	Y44 (CD2)	9.5
	W49 (CZ3)	7.3

### Steady-state fluorescence spectroscopy studies

Steady-state fluorescence emission spectra were collected upon excitation of the Trp pool of the protein at 295 nm. Excitation spectra were acquired with emission wavelength at 330 nm. All measurements were conducted on a fluorescence RTC 2000 spectrometer (Photon Technology International, Canada, Inc.347 Consortium Court London, Ontario N6E 2S8) with a T-configuration, using a 75-W Xenon arc lamp coupled to a monochromator. Samples were analyzed in quartz high precision cell with 10 cm x 2 cm of light path (Hellma Analytics) and gently shaken before each measurement. All slits were set to 5 mm.

### Continuous 295 nm illumination of EGF

#### Temperature effect on EGF photochemistry

**Fig 2:**
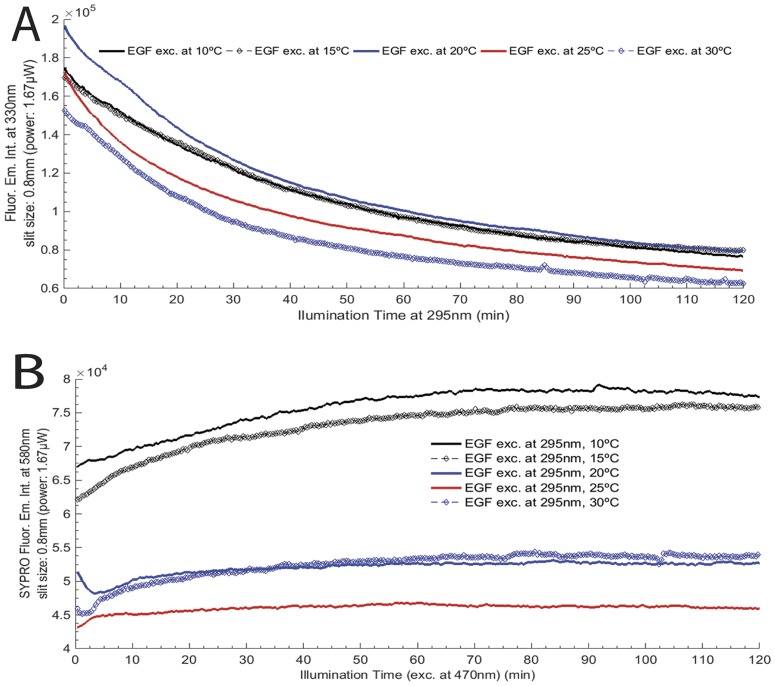
Temperature effect on EGF photochemistry: A) EGF fluorescence excitation and emission wavelengths were fixed at 295 nm and 330 nm, respectively, at 10°C, 15°C, 20°C, 25°C and 30°C; B) SYPRO^®^ Orange fluorescence excitation and emission wavelengths were fixed at 470 nm and 580 nm, at the same temperatures. Continuous illumination was conducted during 2 hours and the excitation slit size was set at 0.8 mm (1.67 μW) for all experiments.

**Fig 3:**
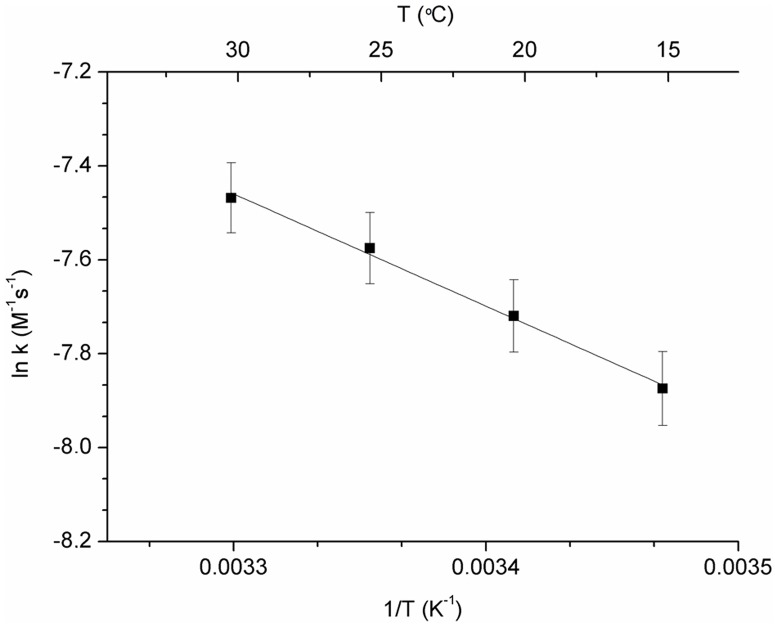
Arrhenius plot showing the linear correlation between the logarithm of the kinetic rate constant (*ln k*) and the inverse of temperature *1/T* (*ln k = ln A*_*0*_*—E*_*a*_*/RT)*, R^2^ = 0.994). The activation energy (*E*_*a*_) and the pre-exponential factor (*A*_*0*_) were 19.9±0.9 kJ.mol^-1^ and 0.44±0.37 M^-1^.s^-1^, respectively. Uncertainty errors for *ln k* values are represented as error bars (percent of data: 1%).

Continuous 295 nm illumination of EGF (fresh sample, 2.5 μM) was carried out for 2 hours and the protein’s fluorescence emission intensity at 330 nm was monitored at five different temperatures: 10°C, 15°C, 20°C, 25°C and 30°C (Fig 2). Excitation slit was set at 0.8 mm, with an equivalent lamp power of 1.67 μW. Fresh samples were used for each experiment. Emission and excitation intensity spectra were corrected in real-time for oscillations in the emission intensity of the excitation lamp. The Arrhenius plot for free EGF was also represented and all parameters calculated, as explained further in the “Data analysis” section (Fig 3).

#### Light power effect on EGF photochemistry

**Fig 4:**
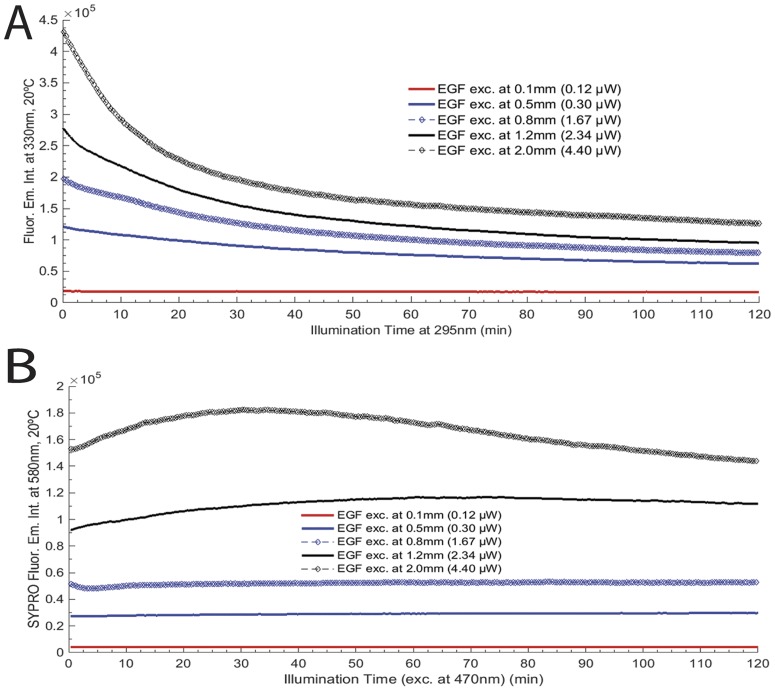
UV-light power effect (different excitation slit openings) on EGF photochemistry: A) EGF fluorescence excitation and emission wavelengths were fixed at 295 nm and 330 nm, respectively, for 0.1 mm (0.12 μW), 0.5 mm (0.30 μW), 0.8 mm (1.67 μW), 1.2 mm (2.34 μW) and 2.0 mm (4.40 μW); B) SYPRO^®^ Orange fluorescence excitation and emission wavelengths were fixed at 470 nm and 580 nm, for the same power levels. Continuous illumination was conducted during 2 hours and the temperature of each solution was kept at 20°C for all experiments.

Continuous 295 nm illumination of EGF (fresh sample, 2.5 μM) was carried out for 2 hours and the peptide’s fluorescence emission intensity at 330 nm was monitored using different excitation slit openings: 0.1 mm, 0.5 mm, 0.8 mm, 1.2 mm and 2.0 mm corresponding to 0.12 μW, 0.30 μW, 1.67 μW, 2.34 μW and 4.40 μW, respectively (Fig 4). Fluorescence excitation (em. fixed at 330 nm) and emission (exc. fixed at 295 nm) spectra of EGF were acquired before and after each EGF illumination using different excitation slit openings. The excitation slit size *versus* excitation power was determined by measuring the power level at the cuvette location with a power meter (Ophir Photonics StarLite Meter ASSY ROHS, P/N7Z01565, Jerusalem, Israel) and a power head (Ophir Photonics, 30A-BB-18 ROHS, P/N7Z02692, Jerusalem, Israel) upon varying the excitation slit size, as previously reported for lysozyme [23]. The temperature of the solution was kept at 20°C using a Peltier element at the cuvette holder location. A fresh sample was used for each illumination session.

#### SYPRO^®^ Orange: probing EGF conformation changes induced by 295 nm and temperature

SYPRO^®^ Orange is used as a molecular probe in order to monitor protein conformational changes since its fluorescence is greatly enhanced upon contact with hydrophobic environments [24]. A 2 μL aliquot (dilution 1:1000) of SYPRO^®^ Orange stock solution (5,000X Concentrate in DMSO) was added to a cuvette containing a fresh sample of EGF (2.5 μM, 0.2 mL) prior to the 295 nm continuous illumination experiment. The sample was gently shaken to mix both solutions. Fluorescence emission of SYPRO^®^ Orange at 580 nm was monitored upon continuous illumination at 470 nm for 2 hours, at each of the above mentioned temperatures, i.e., 10°C, 15°C, 20°C, 25°C and 30°C. Fluorescence intensity changes were quantified. In addition, the fluorescence emission of SYPRO^®^ Orange at 580 nm was monitored upon continuous illumination at 470 nm for 2 hours, at each of the above mentioned power levels, i.e., 0.12 μW, 0.30 μW, 1.67 μW, 2.34 μW and 4.40 μW (corresponding to 0.1 mm, 0.5 mm, 0.8 mm, 1.2 mm and 2.0 mm slits, respectively). Fluorescence spectral changes were quantified.

#### Photoproducts of tryptophan and tyrosine

Fluorescence excitation and emission spectra of the Trp and Tyr photoproducts (e.g., NFK, Kyn and DT) were monitored. Excitation and emission fluorescence spectra of the photoproducts differ from the ones of Trp and Tyr: NFK and Kyn are excited at 320 nm and 360 nm and show a maximum emission between 400–440 nm and between 434–480 nm, respectively [25–27]. Therefore, EGF fluorescence intensity changes and spectral shifts were quantified, before and after the illumination of EGF at 295 nm, at different temperatures and different light power slit openings.

#### Photochemistry of EGF conjugated with HAOA-coated gold nanoparticles

**Fig 5:**
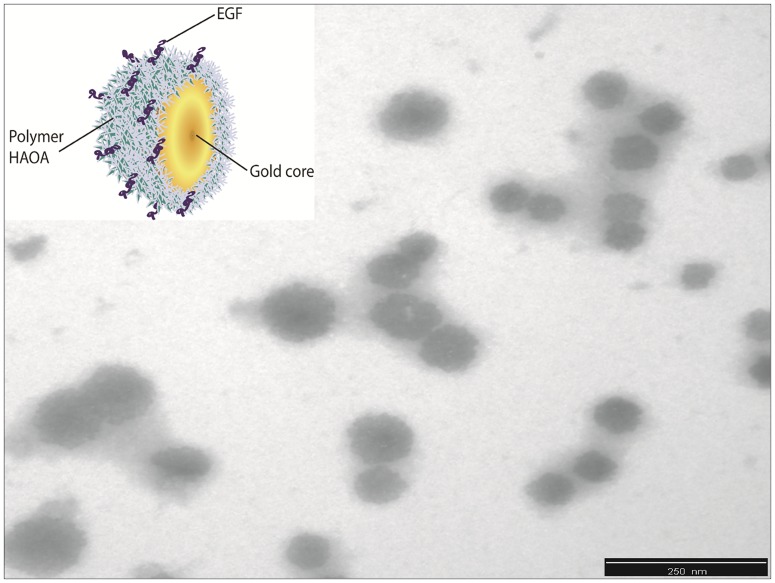
EGF conjugated HAOA-coated gold nanoparticles represented as an illustration (upper corner) and as the TEM image at scale bar of 250 nm.

**Fig 6:**
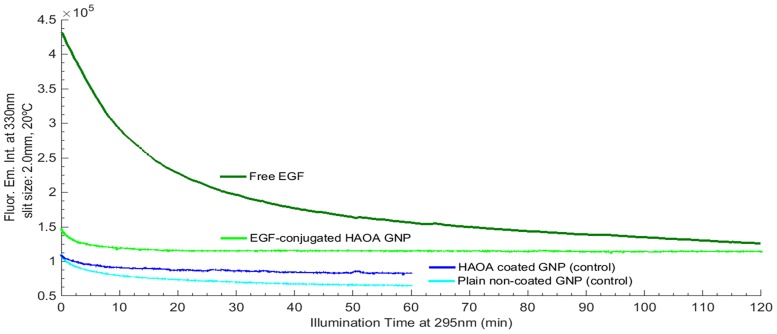
EGF fluorescence emission intensity at 330 nm for free EGF (2 hours 295 nm excitation), EGF-conjugated HAOA-coated gold nanoparticles (2 hours 295 nm excitation), and empty HAOA-coated gold nanoparticles and non-coated plain gold nanoparticles (1 hour 295 nm excitation). All samples were analyzed at 20°C and excitation slit size fixed at 2.0 mm (4.40 μW).

**Fig 7:**
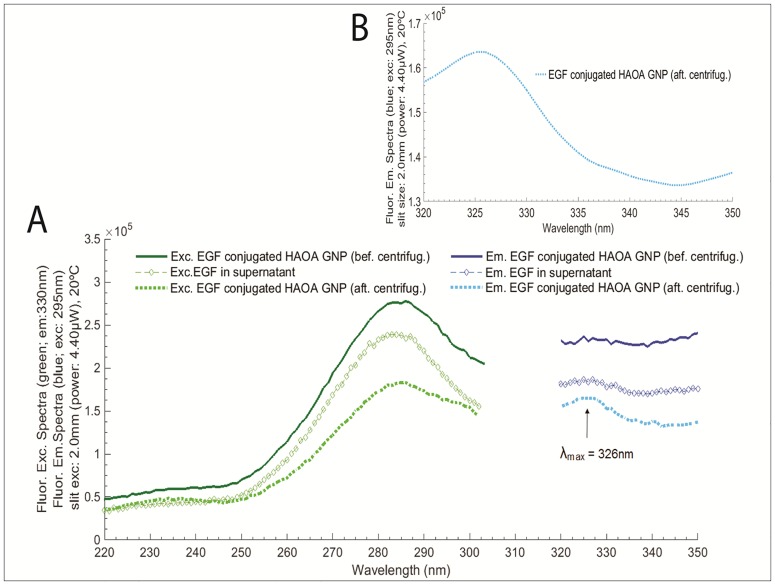
A) Conjugation effect: EGF in supernatant (after conjugation) compared with free EGF and EGF-conjugated HAOA-coated gold nanoparticles; B) EGF-conjugated HAOA-coated gold nanoparticles, at different scale bar. Fluorescence excitation spectra was fixed at 330 nm and fluorescence emission spectra was fixed at 295 nm. Experiments were conducted at 20°C and excitation slit size fixed at 2.0 mm (4.40 μW). No continuous excitation of EGF was conducted, beside the necessary for obtaining the represented spectra.

**Fig 8:**
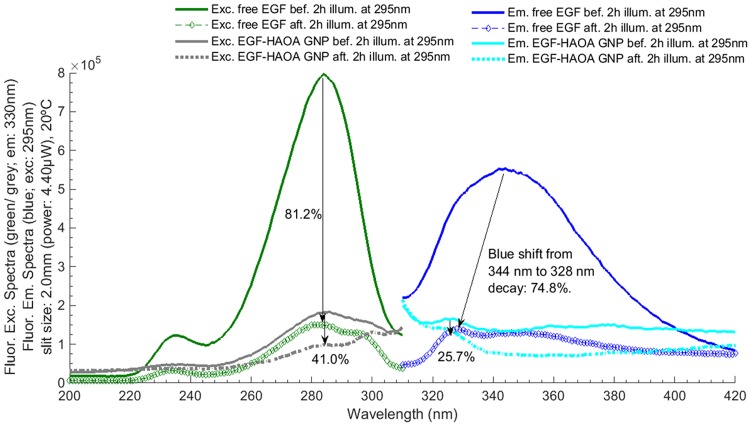
EGF fluorescence excitation and emission spectra acquired before and after 295 nm illumination for 2 hours. Trp fluorescence excitation and emission wavelengths were fixed at 295 m and 330 nm, respectively. Excitation slit size was set at 2.0 mm (4.40 μW) and the temperature of each solution was kept at 20°C for all experiments.

**Fig 9:**
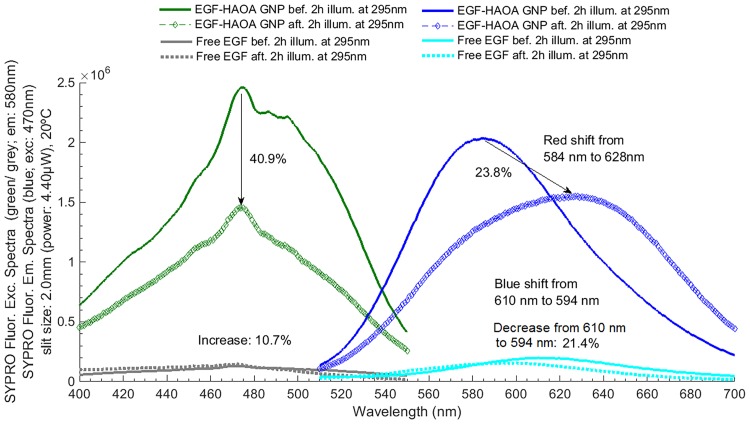
SYPRO^®^ fluorescence excitation and emission spectra acquired before and after EGF 295 nm illumination for 2 hours. SYPRO^®^ Orange fluorescence excitation and emission wavelengths were fixed at 470 nm and 580 nm, respectively. Excitation slit size was set at 2.0 mm (4.40 μW) and the temperature of each solution was kept at 20°C for all experiments.

**Fig 10:**
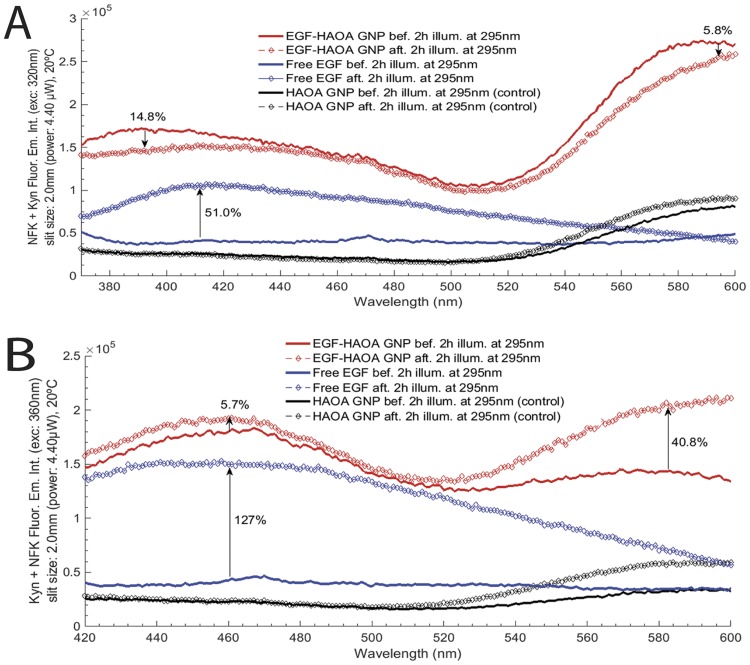
**A)** Fluorescence emission spectra for NFK + Kyn, before and after excitation of free EGF and EGF-conjugated HAOA-coated gold nanoparticles, at a fixed wavelength of 320 nm. Experiments were conducted at 20°C and excitation slit size fixed at 2.0 mm (4.40 μW); **B)** Fluorescence emission spectra for Kyn + NFK, before and after excitation of free EGF and EGF-conjugated HAOA-coated gold nanoparticles, at a fixed wavelength of 360 nm. Experiments were conducted at 20°C and excitation slit size fixed at 2.0 mm (4.40 μW).

The effect of continuous 295 nm excitation of EGF has been investigated for EGF conjugated to gold nanoparticles covered by natural polymers, such as hyaluronic acid (HA) and oleic acid (OA) (Fig 5). Results were compared with data obtained with free EGF. Four samples were continuously illuminated with 295 nm light for 2 hours at 20°C and their fluorescence emission intensity at 330 nm has been monitored: a) free EGF, b) EGF-conjugated HAOA-coated nanoparticles, c) plain non-coated gold nanoparticles and d) HAOA coated-gold nanoparticles (Fig 6). Excitation slit was set to 2.0 mm, with an equivalent power of 4.40 μW at the entrance of the excitation chamber. Conjugation of EGF onto the HAOA-coated gold nanoparticles has been confirmed using steady state fluorescence spectroscopy. Fluorescence excitation (em. fixed at 330 nm) and emission (exc. fixed at 295 nm) spectra of non-conjugated EGF, of the supernatant after centrifugation of the solution containing conjugated and non-conjugated EGF, and of conjugated EGF onto HAOA-coated gold nanoparticles, have been acquired in order to detect the presence of protein (Fig 7). In order to detect likely light-induced conformational changes in EGF, SYPRO^®^ Orange was used as a molecular probe. Fluorescence emission spectra of SYPRO^®^ Orange (excitation fixed at 470 nm) and fluorescence excitation spectra of SYPRO^®^ Orange (emission fixed at 580 nm) were also acquired prior and after continuous illumination of EGF and EGF-conjugated HAOA-coated gold nanoparticles at 295 nm for 2 hours (Figs 8 and 9). Formation of Trp photo products (Kyn and NFK) upon 295 nm excitation of free EGF and EGF-conjugated HAOA-coated gold nanoparticles has been confirmed using steady state fluorescence spectroscopy (Fig 10). In order to detect Kyn and NFK, fluorescence emission spectra were acquired upon 320 nm excitation of the solution before and after 2 hours of continuous illumination at 295 nm. In order to detect the presence of Kyn, emission spectra were obtained upon 360 nm excitation before and after 295 nm continuous excitation. Fluorescence spectral changes have been quantified and compared for free and conjugated EGF. A fresh sample was used for each illumination run.

### Physical characterization of EGF-conjugated HAOA-coated gold nanoparticles

Mean particle size, polydispersity index (PI) and zeta potential (ZP) for EGF-conjugated HAOA-coated gold nanoparticles were determined with a Coulter Nano-sizer Delsa Nano^™^C (Fullerton, CA). A low value of PI factor (< 0.25) will indicate a less dispersed nanoparticles distribution in size. “D-value” was determined as the size distribution in 10%, 50% and 90% of the nanoparticles population [28]. EGF-conjugated HAOA-coated gold nanoparticles were characterized by UV-visible spectroscopy (Evolution 600, UK) and the respective maximum absorbance wavelength (λ_max_) was determined.

### TEM analysis of EGF-conjugated HAOA-coated gold nanoparticles

Structure and surface morphology of EGF-conjugated HAOA-coated gold nanoparticles were analyzed by Transmission Electron Microscopy (TEM, Zeiss M10, Germany) (Fig 5). Samples were prepared through “sequential two-droplet” method by re-suspending the nanoparticles in distilled water and placing a drop (5–10 μL) of the suspension on to a formvar grid for 30–60 sec. When the nanoparticles suspension had partly dried, the surface of the grid was washed three times with distilled water and the excess of water was removed with a filter paper. Then, sodium phosphotungstate (PTA, 2%, w/v) was applied to the grid for 10 sec, the excess of stain removed with a filter paper and the grid was left to dry at room temperature for 24 hours. Samples were analyzed at voltage setting of 10–20 kV. Different fields of the images were recorded digitally.

### Confocal fluorescence microscopy studies with EGF-conjugated HAOA-coated gold nanoparticles

**Fig 11:**
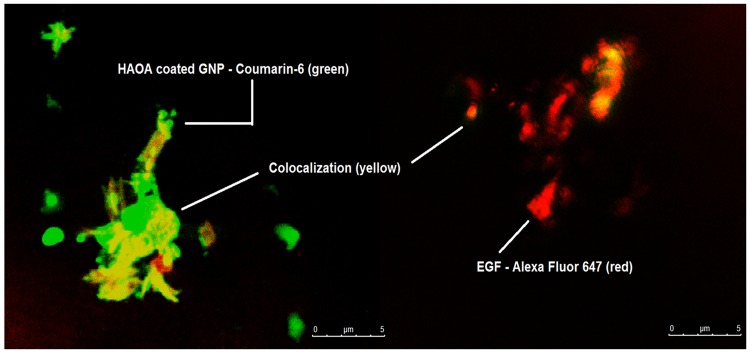
Colocalization of HAOA-coated gold nanoparticles conjugated with EGF. EGF was dyed with Alexa Fluor 647 (red color) and HAOA-coated gold nanoparticles were dyed with Coumarin-6 (green color). The parts were the HAOA-coated gold nanoparticles are associated with EGF, in the same localization, are visible in yellow (scale bar at 5 μm).

EGF-conjugated HAOA-coated gold nanoparticles were marked with two different fluorescent probes, Coumarin-6 and Alexa Fluor 647, as described below, for confocal microscope visualization and colocalization (Fig 11). Firstly, an aliquot (20 μL) of a saturated solution of Coumarin-6 (λ_max_ex_ = 460 nm, λ_max_em_ = 500 nm) in ethanol was added to an aqueous suspension, containing the polymer HAOA and the gold nanoparticles at 1:1 (v/v). Then, EGF marked with Alexa Fluor 647 (λ_max_ex_ = 650 nm, λ_max_em_ = 665 nm) was added to the HAOA-coated gold nanoparticles suspension. Coumarin-6 labeled nanoparticles were allowed to conjugate with the EGF-Alexa Fluor 647 for 30 min at room temperature, and were left 24 hours at 4°C, protected from the light. The suspension was centrifuged twice at 500 x g for 20 min in a FV2400 Microspin (BioSan, Riga, Latvia) to remove unbound EGF. The pellet was re-suspended in PBS buffer (pH 7.4). Confocal Laser Scanning Microscopy (CLSM, Leica, SP5, Mannheim, Germany) was used to verify the colocalization of both dyes on the EGF-conjugated HAOA-coated gold nanoparticles. The chosen excitation laser line He-Ne was 561 nm and the fluorescence emission selected range was set to 569–666 nm. Each sample was analyzed at room temperature and upon letting it dry on a glass slide. Different fields of the images were recorded digitally.

### Circular dichroism spectroscopy

**Fig 12:**
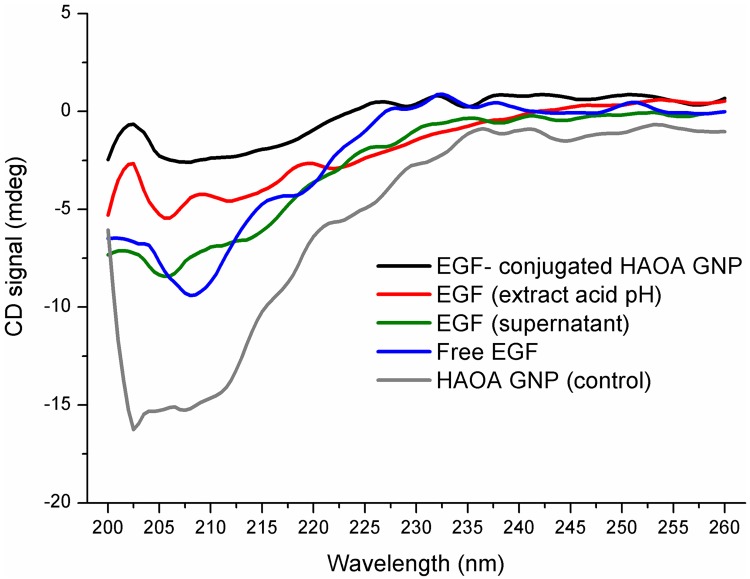
The CD spectra of: free EGF (0.3 mg/mL), HAOA coated gold nanoparticles (without EGF), EGF-conjugated HAOA coated gold nanoparticles (16.5 μg/mL), non- conjugated EGF in supernatant, extracted by centrifugation, and EGF extracted after incubation of EGF-conjugated HAOA coated gold nanoparticles in phosphate buffer pH 5.5, at 37°C, for 72 hours.

Far UV circular dichroism (CD) spectroscopy was carried out to detect any changes in EGF secondary structure after conjugation with HAOA-coated gold nanoparticles using a Jasco J-720 spectropolarimeter (Jasco Corporation, Easton, MD, USA), with a photomultiplier suitable for the 200–700 nm range (Fig 12). After calibration to remove the noise of the device, the PBS buffer used to prepare the native EGF solution and Milli-Q water used for nanoparticles formulations were used as references to obtain the respective baselines. Far UV spectra were acquired using a quartz cell containing solutions of free EGF (0.3 mg/mL), EGF-conjugated HAOA-coated gold nanoparticles (16.5 μg/mL) and HAOA-coated gold nanoparticles (without peptide). Furthermore, spectra of EGF extracted from HAOA-coated gold nanoparticles by two different methods were recorded: 1) EGF non-conjugated present in the supernatant after centrifugation of EGF-conjugated HAOA-coated gold nanoparticles at 7200 x g for 10 min and 2) after incubation of EGF-conjugated HAOA-coated gold nanoparticles in PBS pH 5.5, at 37°C, for 72 hours, followed by centrifugation at 9000 x g for 3 min. Scanning of each sample was conducted from 200 nm to 260 nm with a resolution of 1 nm band width, 3 accumulations, scan speed 100 nm/min and 2 seconds response time. Data was processed using 10 point smoothing in Origin 8.1 (OriginLab Corporation, Northampton, MA, USA).

### Cytotoxicity assays in HaCaT cell line model

**Fig 13:**
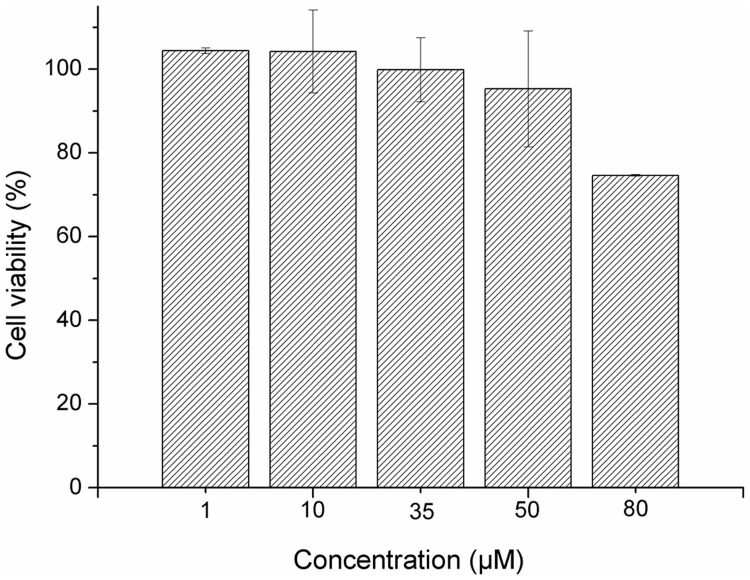
Viability (%) of HaCaT cells exposed to EGF-conjugated HAOA-coated gold nanoparticles for 24 hours, assessed by MTT assay (n = 3–4; mean ± SD).

Cell viability studies were conducted in human immortalized keratinocytes (HaCaT, CLS Cell Lines Service GmbH, Epplheim, Germany) using the MTT assay [29, 30] in order to assess the cytotoxicity of EGF-conjugated HAOA-coated gold nanoparticles (Fig 13). Cells were cultured in Dulbecco’s Modified Eagle Medium (DMEM) medium supplemented with 10% fetal bovine serum (FBS) and 1% penicillin/streptomycin solution. HaCaT cells were seeded onto 96-well plate at a density of 5,000 cells/ well to reach the desired confluence. EGF-conjugated HAOA-coated gold nanoparticles were tested at different concentrations: 0–80 μM (based on the concentration of gold). DMSO 5% (v/v) was used as the positive control. Cells were exposed to nanoparticles for 24 hours. After this period, the cells were washed twice with PBS and incubated with MTT solution (0.5 mg/mL in culture medium) for 2.5 hours at 37°C. Culture medium was then removed and cells were washed again with PBS. DMSO (200 μL per well) was added to dissolve the formazan crystals and absorbance was read at 595 nm (Thermo Scientific Multiskan FC, Shanghai, China). Three to four independent experiments were carried out, each comprising four replicate cultures.

### EGFR binding assay on A549 cells GFP-EGFR

**Fig 14:**
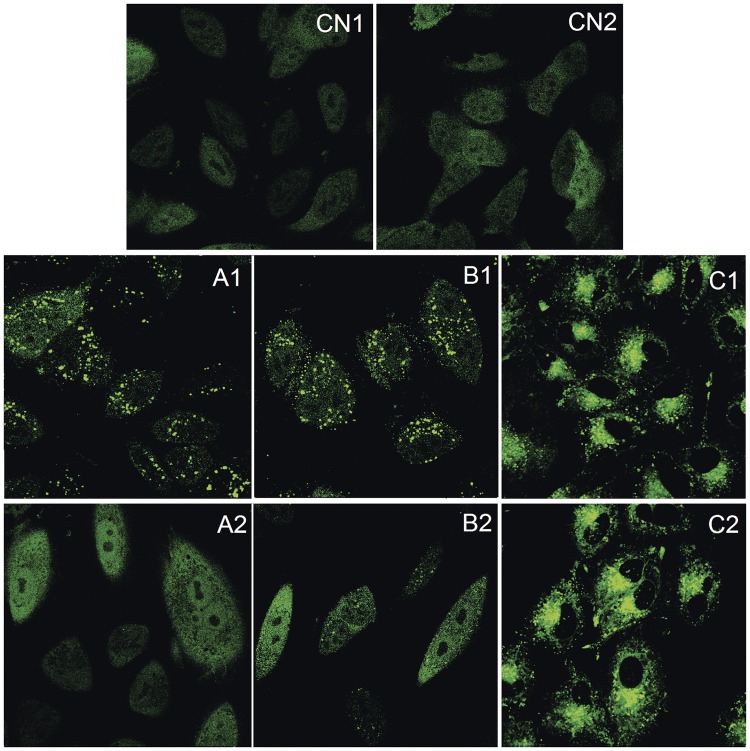
EGFR binding assay in A549 cell model, for 1.5 hours in contact with treatment (100X). CN1 corresponds to the non-treated cells, while CN2 shows the exposure to HAOA-coated gold nanoparticles (without any dye). As for the treatment groups: A1) free EGF with Alexa Fluor 647, B1) EGF-conjugated HAOA-coated gold nanoparticles (only EGF is marked with Alexa Fluor 647), and C1) EGF-conjugated HAOA-coated gold nanoparticles (both EGF and HAOA-coated gold nanoparticles are marked with Alexa Fluor 647 and Coumarin-6, respectively). For A2, B2 and C2, anti-EGFR antibodies were added 1 hour before the addition of the tested samples.

*In vitro* studies were carried out in A549 cells, in which the genomic EGFR gene has been endogenously tagged with a Green Fluorescent Protein gene (GFP) (Sigma-Aldrich ref. CLL1141), since this is a specific and well-studied cell model for EGFR binding assay [30]. A549 cells were cultured in DMEM medium + FBS 10% and 1 μg/mL puromycin and maintained at a 37°C in a 5% CO_2_ atmosphere, in order to analyze the effects of adding free EGF (4 μg/mL), EGF-conjugated HAOA-coated gold nanoparticles (4 μg/mL EGF; 60 μM gold nanoparticles) and HAOA-coated gold nanoparticles (non-conjugated; 60 μM). EGF-conjugated HAOA-coated gold nanoparticles were marked with two different fluorescent probes, Coumarin-6 (λ_max_ex_ = 460 nm, λ_max_em_ = 500 nm) and Alexa Fluor 647 (λ_max_ex_ = 650 nm, λ_max_em_ = 665 nm), as previously described, for confocal microscope visualization and colocalization experiments (Fig 14). Free EGF was marked with Alexa Fluor 647, while nanoparticles were labeled with Coumarin-6. Prior to the image acquisition in the confocal fluorescence microscope, the cells were incubated 1.5 hours with the free EGF and with the EGF-conjugated HAOA-coated gold nanoparticles. In some wells, a primary mouse monoclonal antibody anti-EGFR (1 μg/mL of neutralizer antibody LA1, Millipore (05–101)) was used to block EGFR. After 1 hour of incubation with the antibody, free EGF or EGF-conjugated HAOA-coated gold nanoparticles were added to the A549 cells’ incubation medium, for incubation during 1.5 hours, to see if they compete for the receptor binding and consequent receptor internalization. As controls, non-treated cells and HAOA-coated gold nanoparticles loaded with Coumarin-6 (without EGF) were used. EGFR binding and activation was analyzed by confocal fluorescence microscopy (CLSM, Leica, SP5, Mannheim, Germany). Ligand binding to EGFR activates the receptor and the GFP tagged receptor initially localized on the cell membranes, is then internalized. This leads to the appearance of fluorescence granules in the cell cytoplasm, as described previously for Human EGFR Live Cell Fluorescent Biosensor Assay (Sigma-Aldrich, Germany).

### Data Analysis

All data analysis, plotting and fitting procedures were done using Origin 8.1 (OriginLab Corporation, Northampton, MA, USA).

#### Emission Spectra and Excitation Spectra

Emission and excitation spectra were first smoothed using a 10 points adjacent averaging. All fluorescence spectra obtained were first Raman corrected by subtracting the spectra recorded for the buffer in solution. Normalized emission and excitation spectra were obtained by dividing each data point by the maximum intensity value in each spectrum.

### Fitting Procedures

#### EGF fluorescence emission kinetic traces (em. at 330 nm) upon 295 nm continuous excitation as a function of light power and temperature

Each decay curve acquired upon 2 hours of continuous 295 nm illumination of EGF exposed at different temperatures (10°C, 15°C, 20°C, 25°C and 30°C) and different excitation slit openings (0.5 mm, 0.8 mm, 1.2 mm and 2.0 mm), was fitted using a single exponential decay model given by the function *F(t) = C*_1_*exp(-x*k_*1*_*) + y*_*0*_ or a double exponential decay model, according to *F(t) = y*_*0*_*+C*_1_*exp(-k_1_*x)+C_2_*exp(-k_2_*x). *F(t)* is the fluorescence emission intensity at 330 nm (a.u.) upon 295 nm at excitation time *t* (min), *y*_*0*_, *C*_*1*_ and *C*_*2*_ are constants and *k*_*1*_, *k*_*2*_ are the rate constant of fluorescence emission intensity decrease (min^-1^); *y*_*0*_ value was fixed to 0. Root mean square error R^2^ was > 0.99 for all fitted traces. A double exponential decay model was selected if the single decay model did not provide a good fit. Data obtained with 0.1 mm slit size, was fitted using a linear model (*F(t) = y*_*0*_*+C*_1_*x). A good fit was based on the errors associated to the different parameters and the root mean square error. Fitted parameter values and corresponding errors, and root mean square error values obtained after fitting the 330 nm emission kinetic traces are displayed in Tables 2 and 3.

**Table 2:** Single exponential fit using model *F(t) = C*_1_*exp(-x*k_*1*_*) + y*_*0*_ for each decay curve of EGF at 10°C, 15°C, 20°C and 30°C. For the decay curve of EGF at 25°C, a double exponential fit using model *F(t) = y*_*0*_*+C*_1_*exp(-k_1_*x)+C_2_*exp(-k_2_*x) was selected (see Fig 2A). Fit parameters are displayed in this table. Adj. R^2^ stands for Adjusted R- Square.

Decay	Parameters					Statistic
	y_0_	C_1_	C_2_	k_1_	k_2_	Adj. R^2^
10°C	7.1E+4±1.5E+2	1.0E+6±1.4E+2	-	1.1E+3±4.7	-	0.999
15°C	7.2E+4±1.5E+2	9.8E+4±1.4E+2	-	1.1E+3±6.2	-	0.999
20°C	7.7E+4±2.0E+2	1.2E+5±2.4E+2	-	2.2E+3±1.3E+1	-	0.999
25°C	5.6E+4±3.9E+2	4.3E+4±5.9E+2	7.1E+4±3.1E+2	2.8E+2±4.1	1.7E+3±3.0E+1	0.999
30°C	6.3E+4±1.7E+2	9.0E+4±2.8E+2	-	7.5E+2±5.9	-	0.997

**Table 3:** Single exponential fit using model *F(t) = C*_1_*exp(-x*k_*1*_*) + y*_*0*_ for each decay curve of EGF at a power slit size of 0.5 mm and 0.8 mm (corresponding to 0.30 μW and 1.67 μW, respectively) and double exponential fit using model *F(t) = y*_*0*_*+C*_1_*exp(-k_1_*x)+C_2_*exp(-k_2_*x) for each decay curve of EGF at a power slit size of 1.2 mm and 2.0 mm (corresponding to 2.34 μW and 4.40 μW, respectively) (see Fig 4A). For slit 0.1 mm (0.12 μW), a linear model was selected. Fit parameters are displayed in this table. R^2^ stands for Adjusted R- Square.

Slit(mm)	Decay Parameters					Statistic
	y_0_	C_1_	C_2_	k_1_	k_2_	R^2^
0.1	1.8 E+4±1.1E+1	-0.16±2.7E-3	-	-	-	0.921
0.5	5.7E+4±1.2E+2	6.2E+4±1.0E+2	-	3.0E+4±1.5E+1	-	0.999
0.8	7.7E+4±2.0E+2	1.2E+5±2.4E+2	-	2.2E+3±1.3E+1	-	0.999
1.2	5.8E+4±1.1E+4	9.0E+4±5.6E+3	1.2E+5±5.5E+3	7.9E+3±2.1E+3	1.2E+3±3.7E+1	0.999
2.0	9.8E+4±1.8E+3	2.2E+5±1.3E+3	1.2E+5±6.4E+2	6.8E+2±4.9	5.1E+3±2.1E+2	0.999

#### EGF photochemistry: Arrhenius plot and activation energy

Temperature dependence of the decay constant of the EGF kinetic traces (Fig 2A) (where the fluorescence emission intensity at 330 nm is displayed upon 295 nm excitation), was analyzed using four different temperatures: 15°C, 20°C, 25°C and 30°C. Data was fitted according to the logarithmic form of the Arrhenius equation: *ln k = ln A*_*0*_ *+ (E*_*a*_*/RT)*, where *A*_*0*_ is the pre-exponential factor, *E*_*a*_ is the activation energy, *R* is the universal constant for perfect gases (*R* = 8.314 J/ mol.K) and *T* is the temperature (in Kelvin). The Arrhenius plot and extracted parameters are displayed in Fig 3.

#### Free EGF and EGF conjugated HAOA—coated gold nanoparticles fluorescence kinetics (em. at 330 nm) upon 295 nm excitation

Fluorescence emission intensity kinetic traces at 330 nm for free EGF, plain non-coated gold nanoparticles, HAOA-coated gold nanoparticles and EGF-conjugated HAOA-coated gold nanoparticles samples are displayed in Fig 5. Traces were acquired upon continuous 295 nm illumination for 2 hours, at 20°C, except for plain gold nanoparticles and HAOA-coated gold nanoparticles without EGF, which were illuminated for 1 hour. All traces were fitted using a double exponential decay model according to the formula *F(t) = y*_*0*_*+C*_1_*exp(-k_1_*x)+C_2_*exp(-k_2_*x). *F(t)* is the fluorescence emission intensity at 330 nm (a.u.) upon 295 nm excitation at time *t* (min), *y*_*0*_, *C*_1_ and *C*_2_ are constants and *k*_1_ *and k*_2_ is the rate constant of fluorescence emission intensity decrease (min^-1^); *y*_*0*_ value was fixed to 0. The root mean square error R^2^ was > 0.99 for all kinetics. The fitted parameter values and corresponding errors, and root mean square error values obtained after fitting the 330 nm emission kinetic traces are displayed in Table 4.

**Table 4:** Double exponential fit using model *F(t) = yo+C*_1_*exp(-k_1_*x)+C_2_*exp(-k_2_*x) for free EGF, EGF-conjugated HAOA-coated GNP (gold nanoparticles), plain non-coated GNP (control) and HAOA-coated GNP (control) (see Fig 5). Fit parameters are displayed in this table. Adj. R^2^ stands for Adjusted R- Square.

Samples	Constants (y_0_, k_1_, k_2_)	Adj. R^2^	Pre-exponential factors (C_1_, C_2_)
**Free EGF**	y_0_ = 9.8E+4 ± 1.8E+3 k_1_ = 6.8E+2 ± 4.9 k_2_ = 5.1E+3 ± 2.1E+2	0.999	C_1_ = 2.2E+5 ± 1.3E+3 C_2_ = 1.2E+5 ± 6.4E+2
**EGF—conjugated HAOA GNP**	y_0_ = 1.2E+5 ± 4.4E+2 k_1_ = 4.3E+2 ± 1.8E+1 k_2_ = 3.9E+3 ± 2.3E+2	0.996	C_1_ = 1.8E+4 ± 4.4E+2 C_2_ = 2.2E+4 ± 2.2E+2
**Plain non-coated GNP (control)**	y_0_ = 7.7E+4 ± 1.0E+3 k_1_ = 5.7E+2 ± 5.2E+1 k_2_ = 6.5E+3 ± 5.2E+2	0.997	C_1_ = 6.2E+3 ± 3.7E+2 C_2_ = 2.6E+4 ± 6.6E+2
**HAOA-coated GNP(control)**	y_0_ = 8.9E+4 ± 1.0E+3 k_1_ = 3.5E+2 ± 8.7E+1 k_2_ = 7.6E+3 ± 7.1E+2	0.989	C_1_ = 2.1E+3 ± 2.6E+2 C_2_ = 1.8E+4 ± 8.1E+2

## Results

Although EGF is formed by two amino acid chains (A and B), only chain B is represented in Fig 1. In total, EGF has 2 Trp residues, 5 Tyr residues and 3 SS bridges. Table 1 lists the shortest distances between each Trp and Tyr residues and the nearest SS bonds. The shortest distance between Tyr13 (atom CD1) and the SS bridge C14-C31 is 4.4 Å. All considered distances were < 12 Å. In addition, EGF has no Phenylalanine (Phe) residues but has a considerable number of Arginine (Arg) residues in its structure, close to Trp residues. Arg residues are of considerable importance since they quench the aromatic residues fluorescence emission, when the NH_2_ groups become protonated. The closest distances between these two amino acids occur between Arg45 (NE) and Trp50 (CH2) and Trp49 (CE3) at 4.5 Å and 7.5 Å, respectively.

Firstly, the behavior of the free peptide to temperature and light exposure was assessed. Fig 2A and 2B display the fluorescence kinetic traces for EGF upon 2 hours excitation at 295 nm (emission fixed at 330 nm) and for SYPRO^®^ Orange an analogous experiment (excitation of 470 nm and emission fixed at 580 nm), respectively. At all acquired temperatures, fluorescence emission intensity of Trp is observed to decay as a function of illumination time. On the other hand, fluorescence emission intensity of SYPRO^®^ Orange increases with illumination time. At 10°C and 15°C, EGF showed similar fluorescence emission decays with a decrease in Trp fluorescence emission intensity at 330 nm of 56.2%, 52.8%, respectively. The corresponding increase in the fluorescence emission of SYPRO^®^ Orange after 2 hours excitation of EGF at 295 nm at 10°C and 15°C was 15.6% and 22.7%, respectively. At 20°C and 30°C, the fluorescence emission intensity of Trp decreased 59.6% and 59.1%, respectively, after 2 hours excitation of EGF at 295 nm, while the fluorescence emission intensity of SYPRO^®^ Orange increased 2.3% and 17.3%, respectively. At last, continuous 295 nm excitation of EGF at 25°C led to a 59.7% decrease in the fluorescence emission intensity of the protein and to a 6.7% increase in the fluorescence emission intensity of SYPRO^®^ Orange. When exposing free EGF to five different temperatures, an Arrhenius plot was obtained, as displayed in Fig 3. Due to the temperature dependence of the EGF rate constant (k), recovered from the fluorescence emission decays at 330 nm (excitation at 295 nm), we obtained an activation energy (*E*_*a*_) and a pre-exponential factor (*A*_*0*_) of 19.9±0.9 kJ.mol^-1^ and 0.44±0.37 s^-1^, respectively. The equation obtained was y = -1.1x − 1933.4 (R^2^ = 0.994).

The kinetic traces for free EGF during 2 hours excitation at 295 nm (emission at 330 nm) at 20°C and the kinetic traces for SYPRO^®^ Orange (excitation of 470 nm and emission at 580 nm) using different excitation powers are displayed in Fig 4A and 4B. Excitation of free EGF at 295 nm for 2 hours with different excitation slit sizes of 0.1 mm, 0.5 mm, 0.8 mm, 1.2 mm and 2.0 mm led to a 8.0%, 48.6%, 59.6%, 65.6% and 70.8% decrease in Trp fluorescence emission intensity, respectively. After 295 nm excitation of EGF for 2 hours, the fluorescence emission intensity of SYPRO^®^ Orange increases 9.1%, 2.3%, 21.5% and 6.1% for slit sizes of 0.5 mm, 0.8 mm, 1.2 mm and 2.0 mm, respectively. In the same experiments, the fluoresce emission intensity of SYPRO^®^ Orange has maximally increased by 9.1%, 3.2%, 26.8% and 19.3% for 0.5 mm, 0.8 mm, 1.2 mm and 2.0 mm, respectively. No change was observed in the fluorescence emission intensity of SYPRO^®^ Orange at a slit size of 0.1 mm (decrease: 0.2% = ~0%). A single exponential model (*F(t) = y*_*0*_ *+C*_1_*exp(-x*k_*1*_*)*) was selected to fit the 330 nm decay curves obtained with 0.5 mm and 0.8 mm slit openings. The traces obtained with larger slit openings (1.2 mm and 2.0 mm) were fitted with a double exponential model (*F(t) = y*_*0*_*+C*_1_*exp(-k_1_*x)+C_2_*exp(-k_2_*x)). The corresponding fitted parameter values (C_1_, C_2_ k_1_, k_2_, y_0_) and corresponding errors, as well as root mean square error values, are displayed in Table 3. The 330 nm fluorescence decay obtained when a slit 0.1 mm was chosen was best fitted by a linear model.

After studying the temperature and power dependence of the kinetic traces for free EGF, the behavior of this peptide has been monitored after conjugation with a nanosystem made of a gold core and a biodegradable polymeric coating of hyaluronic and oleic acids (HAOA). HAOA-coated gold nanoparticles (i.e., non-conjugated with EGF) showed a mean particle size of 300 nm (PI: 0.2) and a negatively charged surface (-19 mV) [24]. After conjugation with EGF, the volume distribution for 90% of HAOA-coated gold nanoparticles (D 90%) was 220 nm, as confirmed by TEM analysis, where EGF-conjugated HAOA-coated gold nanoparticles showed a size around 100–200 nm and a spherical morphology (see Fig 5). EGF-conjugated HAOA-coated gold nanoparticles are composed by a dense gold core observed in the TEM image as a dark core, and by a soft polymeric coating of HAOA on the surface, visible in the TEM image as a grey area around the core. EGF may be associated to the HAOA coating of the gold nanoparticles as illustrated in Fig 5. Zeta potential (ZP) of EGF-conjugated HAOA-coated gold nanoparticles was around -5 mV when compared to the lower value of -19 mV for the HAOA-coated gold nanoparticles alone. In addition, a maximum absorbance peak at 655 nm compared to 800 nm observed for the plain non-coated gold nanoparticles, indicating that a 145 nm blue shift has occurred after conjugation.

The EGF fluorescence emission intensity at 330 nm during 2 hours of continuous 295 nm excitation is displayed in Fig 6 and compared for free EGF, EGF-conjugated HAOA-coated gold nanoparticles, empty HAOA—coated gold nanoparticles and non-coated plain gold nanoparticles. Plain gold nanoparticles (i.e., without HAOA coating) and HAOA-coated gold nanoparticles were used as controls. The double exponential fit model (*F(t) = yo+C*_1_*exp(-k_1_*x)+C_2_*exp(-k_2_*x)) used to fit the kinetic traces for free EGF and EGF-conjugated HAOA-coated gold nanoparticles showed that the fluorescence emission intensity of free EGF decayed faster than the one for conjugated EGF with HAOA-coated gold nanoparticles. Decay constants for conjugated EGF were 1.5-fold (k_1_) and 1.3-fold (k_2_) lower compared to the ones for free EGF. Also, the initial Trp 330 nm fluorescence emission intensity (excitation at 295 nm) for EGF-conjugated HAOA-coated gold nanoparticles is almost three times lower than the initial fluorescence emission intensity of free EGF. Fitting results are represented in Table 4.

Afterwards, the effect of conjugation on the fluorescence spectra of EGF was investigated. Fluorescence excitation spectra (emission fixed at 330 nm) and fluorescence emission spectra (excitation fixed at 295 nm) were compared for free EGF in supernatant, EGF-conjugated HAOA—coated gold nanoparticles (before centrifugation) and EGF-conjugated HAOA—coated gold nanoparticles (after centrifugation) (see Fig 7A). Centrifugation at 500 x g for 20 min was essential for the elimination of the non-conjugated EGF and EGF was only illuminated with the light necessary for obtaining the represented spectra. Isolated EGF-conjugated HAOA—coated gold nanoparticles (after centrifugation) showed a clear emission peak at 326 nm, which confirms the presence of Trp residues at the HAOA—coated gold nanoparticles’ surface (see Fig 7B).

Figs 8 and 9 display the fluorescence excitation and emission spectra of EGF, as free peptide and as conjugated with HAOA-coated gold nanoparticles, and of SYPRO^®^ Orange. The fluorescence emission and excitation intensity of SYPRO^®^ Orange is 10 and 18.6 higher, respectively, when added to EGF-conjugated HAOA-coated gold nanoparticles than when added to free EGF. Fluorescence emission intensity of free EGF at 328 nm decreased 74.8%, after illumination, and a blue shift occurred from 344 nm to 328 nm, while the fluorescence emission intensity of EGF-conjugated HAOA-coated gold nanoparticles at 347 nm decreased 25.7%. As for the fluorescence emission intensity of SYPRO^®^ Orange, the values decreased 21.4% and 23.8% after illumination of both free EGF and EGF-conjugated HAOA-coated gold nanoparticles, respectively. Interestingly, the fluorescence emission spectra of SYPRO^®^ Orange showed a blue shift (from 610 nm to 594 nm) when added to free EGF, while when added to EGF-conjugated HAOA-coated gold nanoparticles, the peak of SYPRO^®^ Orange emission spectra showed a red shift from 584 nm to 628 nm (see Fig 9).

In order to detect the putative presence of photochemical species such as NFK and Kyn, fluorescence emission spectra upon 320 nm excitation were acquired for free EGF and for EGF-conjugated HAOA-coated gold nanoparticles, before and after 295 nm continuous illumination of the samples (see Fig 10A). HAOA-coated gold nanoparticles spectra, before and after 2 hours illumination at 295 nm, were used as controls. For free EGF, a peak centered at 418 nm was observed upon 320 nm excitation. The fluorescence emission intensity of the peak increases 51.0% after continuous excitation with 295 nm for 2 hours. For EGF-conjugated HAOA-coated gold nanoparticles, two peaks were observed: a peak centered at 392 nm and a larger peak at 598 nm. The second peak at 596–598 nm is also visible for the controls HAOA-coated gold nanoparticles, without EGF, before and after continuous illumination, though 3 to 4 times less intense. After continuous excitation with 295 nm for 2 hours, the fluorescence emission intensity decreased by 14.8% and 5.8%, for the peak centered at 392 nm and 598 nm, respectively. In Fig 10B are displayed the fluorescence emission intensity spectra upon 360 nm excitation in order to detect the putative presence of the photochemical species Kyn and NFK. HAOA-coated gold nanoparticles spectra, before and after 2 hours illumination at 295 nm, were used as controls. For free EGF, a peak centered at 460 nm is observed upon 360 nm excitation. The fluorescence emission intensity of the peak increases 127% after continuous excitation with 295 nm for 2 hours. Two emission peaks were observed for EGF-conjugated HAOA-coated gold nanoparticles: a peak centered at 461 nm and a larger peak at 580 nm. The second peak at 580 nm is also visible for the control HAOA-coated gold nanoparticles, without EGF, before and after continuous illumination, but with less intensity, like observed upon 320 nm excitation. After continuous excitation with 295 nm for 2 hours, the fluorescence emission intensity decreased by 5.7% and 40.8%, for the peak centered at 461 nm and 580 nm, respectively.

In order to characterize the binding of EGF to HAOA-coated gold nanoparticles, colocalization experiments were conducted in a confocal microscope (Fig 11; scale bar at 5 μm). EGF labeled with Alexa Fluor 647 appears in red and HAOA-coated gold nanoparticles labeled with Coumarin-6 appear in green; EGF-conjugated HAOA-coated gold nanoparticles is displayed in yellow. On the other hand, circular dichroism (CD) is a good method to evaluate changes in the secondary structure of proteins, after binding. Fig 12 shows far UV CD spectra collected for different samples. The spectra show that after conjugation with HAOA-coated gold nanoparticles EGF maintains its secondary structure. Although free EGF (non conjugated) has a signal of higher intensity than the rest of the studied samples (i.e., EGF-conjugated HAOA-coated gold nanoparticles, EGF in supernatant and extracted EGF with acidic pH solution), its concentration was also 18 times higher. The CD spectra indicate that EGF probably has a secondary structure, with contributions from different secondary elements. This is suggested by the presence of a negative peak around 208–210 nm, characteristic of α-helix structure. However, the negative band at 220 nm, also characteristic of α-helix structure was not detected. The absence of CD bands above 215–220 nm range suggests the presence of EGF’s β- sheets.

Finally, cell culture experiments allowed us to understand how the nanoparticles interact with *in vitro* biological systems. When exposing human keratinocytes (HaCaT) to EGF-conjugated HAOA-coated nanoparticles for 24 hours, no aggregates were visible after addition of the nanoparticles to the plaque wells (Fig 13). In addition, EGF-conjugated HAOA-coated gold nanoparticles at 80 μM the highest concentration tested, showed a cell viability of around 75% of that of non-treated control cultures. As for the experiments for the EGFR binding assay carried out with human lung carcinoma A549 cells, images were taken 1.5 hours after the cells being in contact with EGF and for the negative control (Fig 14). Three different samples were tested: free EGF labeled with Alexa Fluor 647 (A1); EGF-conjugated HAOA-coated gold nanoparticles, being EGF labeled with Alexa Fluor 647 (B1); and EGF-conjugated HAOA-coated gold nanoparticles, with EGF labeled with Alexa Fluor 647 and the HAOA-coated gold nanoparticles labeled with Coumarin-6 (C1). In addition, the same samples were tested after the cells were incubated for 1 hour with anti-EGFR antibody, in order to block the EGF receptors. Finally, two control groups were studied: CN1, corresponding to cells from the non-treated group (i.e., cells without the addition of EGF or nanoparticles and of the anti-EGFR antibody) and CN2, corresponding cells in presence of HAOA-coated gold nanoparticles (without dye or EGF conjugation). In panels A1, B1 and C1 it can be observed that EGF has induced EGFR internalization, alone and when conjugated with the HAOA-coated gold nanoparticles. Both free EGF and EGF-conjugated HAOA-coated gold nanoparticles (panels B1 and C1) entered the cells’ cytoplasm but not its nucleus. The anti-EGFR antibody blocked the binding of EGF to EGFR, preventing receptor internalization (panel A2); however, the EGF-conjugated HAOA-coated gold nanoparticles could still enter the cells (panels B2 and C2) despite the presence of the antibody. The controls (panels CN1 and CN2) confirm that in the absence of EGF and in the absence of nanoparticles there is no EGFR activation.

## Discussion

The presented data has shown that the structure of EGF (Fig 1) can be modulated by UV-light (295 nm) and that the photochemical changes are reduced when EGF is bound to HAOA-coated gold nanoparticles (Fig 6). Like other small proteins and peptides (e.g., cutinase, insulin, α-lactoalbumin) [14,17,31], EGF is an interesting model protein for photostability studies due to the close spatial proximity between its aromatic residues and its disulphide (SS) bridges. SS bridges are key structural elements in small proteins [31], responsible for maintaining the proteins´ structure and therefore their function. Disruption of SS bonds induced by UV excitation of aromatic residues in those peptides will most likely destroy its structure and impair its function [14,17,31]. Table 1 lists the three SS bonds of EGF located in close spatial proximity to aromatic residues (Trp and Tyr). The observed close distances will allow for electron transfer between the aromatic residues and the SS bridges [17], leading to the disruption of such bridges. EGF is a protein where these reactions will occur in the presence of UVB light leading to protein conformational changes and to loss of functionality. EGF is a natural ligand for EGFR with significant biomedical importance in cancer treatment and diagnostic [19]. Changes in the fluorescence spectra of the extrinsic fluorescence probe SYPRO^®^ Orange confirmed structural changes of EGF induced by temperature (Fig 2), prolonged illumination at 295 nm (Fig 4) and pH, as its fluorescent emission is enhanced upon binding to hydrophobic regions of the protein [20]. Temperature-dependent time based photochemical studies (see Fig 2A) show that EGF photochemistry and protein conformational space is temperature dependent, being similar at 10°C and 15°C, at 20°C and 30°C but distinct at 25°C. At 25°C, SYPRO^®^ Orange appears to bind less to the peptide than at other temperatures, indicating that EGF has fewer hydrophobic surfaces exposed to the solvent. The Arrhenius plot (see Fig 3) showed that the activation energy (*E*_*a*_) associated with the photochemical reactions induced by 295 nm on EGF was 19.9±0.9 kJ.mol^-1^ (i.e., 4.76 kcal/mol). This value was similar to the one found for α-lactalbumin (E_a_ = 21.8±2.3 kJ.mol^−1^) [31]. Power-dependent irradiation studies of EGF (see Fig 4A) reveal that the larger the power used, the faster the kinetics associated with the fluorescence decays. A single exponential model was used to fit the Trp decay curves acquired with 0.30 μW and 1.67 μW (see Table 3) but for larger powers (2.34 μW and 4.40 μW) a double exponential model was needed. This shows that different photochemical processes are initiated at higher powers when compared to lower powers. Experiments carried out with SYPRO^®^ Orange (see Fig 4B) show the same trend. Conformational changes induced in EGF are larger when illumination was carried out with higher powers: when using a 2.0 mm slit size opening, the fluorescence emission intensity of SYPRO^®^ Orange was higher than when working with a 0.1 mm slit, indicating that the extrinsic probe is in contact with a larger hydrophobic surface rendered accessible due to light induced conformational changes.

Conjugation of EGF to HAOA-gold nanoparticles protected EGF from photochemistry (Fig 6, Table 4): the presence of the particles decreased the rate of the light induced fluorescence changes and induced quenching. Both HA, OA and gold are known to be fluorescence quenchers. EGF has a promising therapeutic value as a targeting ligand for tumours overexpressing EGFR, such as melanoma [32]. Therefore, it has been coupled to nano-sized delivery systems, made of either and both metallic and polymeric materials [33,34]. The photochemical protection conferred by nanoparticulate carriers is advantageous.

Gold nanoparticles were prepared according to a seed-growth method [21,35]. An aqueous extract of *Plectranthus saccatus* (Benth.), rich in anti-oxidative compounds (e.g., rosmarinic acid, caffeic acid and chlorogenic acid [36]), was used as the main reducing and capping agent. Furthermore, a coating made of hyaluronic and oleic acids (HAOA) was added to the gold nanoparticles. Natural polymers can work as reducing and capping agents, activating “green” reduction of gold and being less toxic for healthy tissues, which make them advantageous in the reduction and morphology of gold nanoparticles [35,37]. Furthermore, the use of polymeric coatings is also interesting as a way to control drug release and to increase the adsorption of ligands. Recently, Su et al. (2014) showed that hyaluronic acid (HA) scaffolds can increase the adsorption and sustained release of EGF, attached to the polymeric surface through self-assembly and electrostatic interactions [38]. In addition, HA is reported to confer structural stability to proteins [12]. Herein, we studied the effect of mounting EGF to HAOA-coated gold nanoparticles. In Fig 5, it can be observed that EGF-conjugated HAOA-coated gold nanoparticles show the gold core (dark core) surrounded by HAOA polymer. On the upper left corner of Fig 5 is displayed a model of the EGF-conjugated HAOA-coated gold nanoparticles. Kinetic data displayed in Table 4 and Fig 6 confirms that the polymers and the gold core promoted protein quenching and induced slower decay kinetics when compared with the data obtained with free EGF, protecting EGF from photochemistry. The same has been reported by Oliveira Silva *et al*. (2015) when studying the photochemistry of free lysozyme and comparing it to the photochemistry of lysozyme mounted onto HAOA-coated gold nanoparticles [23]. It is likely that the structure of EGF, after conjugation to HAOA-coated nanoparticles, sustains excitation at 295 nm for longer time periods, prior to possible loss of structure and function. The presence of EGF conjugated to the HAOA-coated gold nanoparticles was confirmed by fluorescence spectroscopy, after centrifugation and re-suspension with PBS, demonstrating a clear emission peak at 326 nm for Trp residues (Fig 7). The conjugation of EGF onto HAOA-coated gold nanoparticles conferred enhanced photostability to EGF. This is observed in Fig 8, where a smaller intensity reduction occurs in both fluorescence excitation spectra (41.0% *versus* 81.2%) and fluorescence emission spectra (25.7% *versus* 74.8%), after 295 nm continuous illumination. Furthermore, the observed blue shift in the fluorescence emission spectra of free EGF, after 295 nm continuous illumination, is no longer visible for EGF-conjugated HAOA-coated gold nanoparticles (Fig 8). This is probably due to the fact that conjugation of EGF has prevented conformational changes that rendered the Trp moieties more apolar, responsible for the blue shift. SYPRO^®^ Orange was used as an extrinsic probe for monitoring UV-light (295 nm) induced conformational changes in EGF (Fig 9). Firstly, it was observed that SYPRO^®^ Orange showed affinity towards hydrophobic moieties in HAOA-coated gold nanoparticles, leading to an increase of its fluorescence emission intensity signal compared to its fluorescence emission intensity in free EGF solution (Fig 9). Secondly, the fluorescence emission spectra of EGF-conjugated HAOA-coated nanoparticles suffered a red shift (from 584 nm to 638 nm) after 2 hours of continuous 295 nm illumination. On the other hand, the fluorescence emission spectra of free EGF suffered a blue shift (from 610 nm to 594 nm) after 2 hours of continuous 295 nm illumination. The observed red shift for SYPRO^®^ Orange reveals that the probe is in a more polar environment after illumination.

Finally, the antioxidant compounds of *Plectranthus saccatus*, present in the HAOA-coated nanoparticles formulation, can also have an important role in protecting EGF from light induced reactions. Phenolic compounds are highly present in natural plant extracts and are described to show anti-oxidant effects on Trp oxidation and to be fluorescence quenchers [39]. It has been shown that the oxidation of the indole ring of Trp can be inhibited and, consequently, the formation of NFK and Kyn, by associating proteins with phenolic compounds from natural plant extracts [39, 40]. This is positively correlated with our data. Conjugation of EGF to HAOA-coated gold nanoparticles reduced or even avoided the formation of photoproducts such as NFK and Kyn (see Fig 10A and 10B).

The presence of oxidative conditions induced by light can lead to the oxidation of the aromatic residues in proteins [14,15,19,31,41]. UVB excitation of aromatic residues in proteins leads to the disruption of SS bridges [14–17,19] and to the formation of photoproducts, such as N-formylkynurenine (NFK), kynurenine (Kyn) [25,42] and dityrosine (DT) [26]. Since 295 nm excites specifically Trp residues, it is very likely that the photoproducts formed are Trp derivatives such as NFK and Kyn and not Tyr derivatives like DT. Furthermore, the emission spectrum of EGF upon 295 nm leads to a fluorescence emission spectrum that peaks around 330 nm, which makes it unlikely that Tyr residues will be excited by EGF emission. Two excitation wavelengths were used in order to detect the presence of photochemical products: 320 nm (Fig 10A) and 360 nm (Fig 10B). Light at 320 nm excites both NFK (ε_NFK(321nm)_ = 3750 M^-1^cm^-1^) [42–45] and Kyn (ε_Kyn(321nm)_ = 1812 M^-1^cm^-1^) [46]. At 315 nm DT has an extinction coefficient equal to 5200 M^-1^cm^-1^ but, as explained above, it is unlikely that it has been formed [47,48]. Light at 360 nm excites NFK (ε_NFK(360nm)_ = 1607 M^-1^cm^-1^ [46] and Kyn (ε_Kyn(365nm)_ = 4530 M^-1^cm^-1^ [49,50]) but does not excite DT. In Fig 10A, the peak with maximum fluorescence emission intensity (320 nm excitation) for free EGF occurs at 418 nm and for EGF-conjugated HAOA-coated occurs at 392 nm. In Fig 10B, the peak with maximum fluorescence emission intensity (360 nm excitation) for free EGF occurs at 460 nm and for EGF-conjugated HAOA- coated nanoparticles it is seen at 461 nm. This peak cannot belong to DT, since DT is not excited at 360 nm. Therefore, it can be Kyn since the wavelength of maximum fluorescence emission of Kyn lies within 434–480 nm.

Colocalization experiments carried out with confocal fluorescence microscopy (Fig 11) confirmed that EGF (red colour) appears to be associated and colocalized with HAOA-coated gold nanoparticles (green colour), which can be visualized as yellow coloured spots. Since EGF shows a pI around 4.55 and HAOA-coated gold nanoparticles have a superficial negative charge (-19 mV), attractive electrostatic interaction between the protein and the nanocarrier are not likely to occur at pH 7.4. In spite of this, a slight increase of the nanoparticles’ surface charge after EGF conjugation (-5 mV) is observed and, as already mentioned, the peptide conjugation onto the particles has also been confirmed by fluorescence spectroscopy. Moreover, literature described that EGF is likely to be associated to hyaluronic acid (HA) scaffolds through the polymer’s carboxylic groups and since HA is a hydrogel with high hygroscopic character, interactions between EGF and the polymer can occur by hydrophilic interactions [12,38,51]. Another possible mechanism for EGF conjugation onto HAOA-coated gold nanoparticles is by means of binding between an amino acid residue of the peptide and the HAOA coating or gold core. Histidine has been described as a very strong metal binding amino acid [52]. EGF has two histidine residues (His10 and His16). Lysine residues (Lys28 and Lys48) of EGF were also pointed out as a potential binding site for EGF conjugation with HA polymer [53], especially Lys48, which is located at the end of lateral chain of EGF.

Far UV spectra for CD showed that EGF maintained its non-helical, random coil structure, before and after conjugation and after extraction from the HAOA-coated gold nanoparticles, when incubated at 37°C in pH 5.5 phosphate buffer (Fig 12). The native structure of EGF is described to be mainly composed of random coil elements (72%) and β-helical elements (25%) and only a trace of α-helical content [3]. The random coil secondary structure contributes to the presence of a negative peak at 200–210 nm [54]. Although a typical shoulder formation at 220 nm is described for EGF [54], as indicative of the presence of random non-helical forms and β-sheets, spectra with a flat curve around 215–225 nm is also expected for EGF, suggesting a low content on β forms [55]. It has been observed this flat curvature for all spectra of EGF samples above 215–220 nm (Fig 12). EGF-conjugated HAOA-coated gold nanoparticles showed a negative peak at 208 nm, as free EGF in its native form, in PBS pH 7.4. However, a spectral blue shift was detected for both EGF in supernatant, as for the unbound peptide, recovered after centrifugation of the EGF-conjugated HAOA-coated nanoparticles and EGF extracted after incubation of EGF-conjugated HAOA-coated gold nanoparticles in phosphate buffer pH 5.5, at 37°C, for 72 hours. Both EGF in the supernatant and extracted EGF’s peaks shifted to 206 nm. Furthermore, HAOA-coated gold nanoparticles, without EGF, showed an intense signal for the main negative peak at 200–210 nm, with a minimum at 201 nm, which can be due to the fact that HAOA coating of gold nanoparticles, especially HA, also absorb in the far UV range [56].

After characterizing the EGF-conjugated HAOA-coated gold nanoparticles in terms of pharmaceutical technology and protein stability, its potential biological application was evaluated. Firstly, the cell viability in normal-like human keratinocytes (HaCaT cell line) was tested in order to verify if our nanoparticles were safe when in contact with a healthy non-cancer tissue (see Fig 13). Statistical analysis was based on Student’s t-test for comparisons between cell viability values with non-conjugated HAOA-coated gold nanoparticles [21] and EGF-conjugated HAOA-coated nanoparticles (present work). No significant differences (p > 0.05) were found between the viability of HaCat cells treated with HAOA-coated gold nanoparticles and EGF-conjugated HAOA-coated gold nanoparticles at equal concentrations. Finally, when testing the biologic activity of EGF-conjugated HAOA-coated nanoparticles, compared to free EGF, for EGFR binding, we have used a well-studied cell model for described previously for Human EGFR Live Cell Fluorescent Biosensor Assay [30]. Therefore, it was observed that both peptides, in those different conditions, were able to bind to the receptor and activate its internalization (visible green fluorescence as shown in Fig 14), making possible downstream signal transduction. It has been observed in our study that the antibody anti-EGFR competitively inhibits the binding of free EGF to EGFR, but not for EGF-conjugated to HAOA-coated gold nanoparticles. Therefore, nanoparticles can also enter the A549 cells by a putative different internalization mechanism than EGFR-modulated internalization. EGF-conjugated HAOA-coated gold nanoparticles may modulate the endocytic pathways for entering the cells. One example is the use of other cell receptors, such as CD44, for which hyaluronic acid is a specific ligand, which are also described to be also overexpressed in many solid tumor cells including breast, melanoma and lung cancer, like the A549 cell model used in this study [57,58]. Qhattal et al. (2011) confirmed this possibility when they demonstrated by fluorescence microscopy that HA-coated liposomes, made of high-molecular weight HA, led to increased uptake of liposomes by A549 cells, with high and irreversible binding affinity [59]. As observed in Fig 14, EGF-conjugated HAOA-coated gold nanoparticles accumulate around the peri-nuclear area, but do not penetrate into the cell nucleus, after 1.5 hours-incubation. Wang et al. (2013) showed that lipid-coated gold nanoparticles promoted the formation of acidic compartments, which appear to be lamellar bodies, in A549 cells [60]. These vesicles appear to internalize the nanoparticles, allowing them to enter the cell. Wang et al. (2013) also state that internalization occurs as a result of the negative charge of gold nanoparticles penetrating the lung surfactant, which primarily contains a mixture of phosphatidyl choline and phosphatidyl glycerol lipids [60]. Other polymeric coated gold nanoparticles, also highly negatively charged (-40 mV) were retained in the endolysosomal compartments, also predominant presence at the perinuclear region, after 1 and 2 hours-incubation with A549 cells [61]. This internalization was reported to occur very quickly, around 1 hour to 2 hours, probably due to EGF small size and high affinity to EGFR. Maybe, as a consequence, the clearance is also faster in lysosomes and in cells with high expression of EGFR [62]. Finally, another study using A549 lung cancer cells, sulfhydryl-activated EGF conjugation with lipidic nanoparticles were colocalized with the labeled EGF receptors and the internalization of EGF-conjugated nanoparticles was visible [33].

In conclusion, the EGF-conjugated HAOA-coated gold nanoparticles developed in this work show a potential application for near infrared (NIR, 650–800 nm) photothermal therapy, which may efficiently destroy cancer cells. NIR photothermal therapy reduces the damage of the healthy tissue compared to visible photothermal therapy, as the optical radiation is the lowest absorbed by the human tissue [1]. HAOA-coated gold nanoparticles protected EGF from 295 nm induced photochemistry and did not induce EGF denaturation, reducing the formation of photoproducts such as NFK and Kyn. Moreover, EGF-conjugated HAOA-coated nanoparticles did not markedly decrease HaCaT cell viability, showing high biocompatibility with healthy tissues, and were able to enter the EGFR-overexpressing tumor cell line A549, by different internalization mechanisms. As future prospects, cancer treatment could benefit from a combined approach, using multiple targeting moieties, for a specific cancer cell pool and acting by several molecular pathways. Another advantage would be the conjugation of a light-absorbing core, for photothermal therapy, and a polymeric coating, capable of promoting the incorporation of anticancer drugs, for a local chemotherapy.
